# Off-Label Use of Antineoplastic Drugs to Treat Malignancies: Evidence From China Based on a Nationwide Medical Insurance Data Analysis

**DOI:** 10.3389/fphar.2021.616453

**Published:** 2021-04-08

**Authors:** Guoxu Wei, Min Wu, He Zhu, Sheng Han, Jing Chen, Chenchen Zhai, Luwen Shi

**Affiliations:** ^1^ Department of Pharmacy Administration and Clinical Pharmacy, School of Pharmaceutical Sciences, Peking University, Beijing, China; ^2^ International Research Center for Medicinal Administration, Peking University Health Science Center, Beijing, China

**Keywords:** off-label use, antineoplastic drugs, malignancies, nationwide medical insurance data, retrospective study

## Abstract

**Purpose:** Cancer is a leading cause of morbidity and mortality worldwide. Off-label (OL) use of antineoplastic drugs to treat malignancies is prevalent. In this study, we quantified and characterized OL use of antineoplastic drugs to treat malignancies in China.

**Methods:** This was a retrospective study using nationwide data collected from 2008 to 2010. Use of antineoplastic drugs was considered OL if they were used for indications not reflected in the package insert published by the National Medical Products Administration at the time of prescription. Descriptive analysis and Spearman rank correlation were used to evaluate the frequency and pattern of OL drug use.

**Results:** In total, 51,382 patients with malignancies, 24 categories of antineoplastic drugs, and 77 types of malignancies treated with OL drugs were included in this study. Twenty commonly used antineoplastic drugs (ICD encoded as L01) were used OL in 10–61% of cases, and four commonly used endocrine therapy antineoplastic drugs (ICD encoded as L02) were used OL in 10–19% of cases. There was a significant negative correlation between the disease constituent ratio and the average OL use rate of antineoplastic drugs for various malignancies. In contrast, there was a significant positive correlation between the average OL use rate of antineoplastic drugs and the number of malignancies treated with OL drugs.

**Conclusion:** This study provided information regarding OL use of antineoplastic drugs for treatment of malignancies, and showed that OL use was prevalent. In addition, uncommon malignancies were more likely to be treated with OL antineoplastic drugs. Furthermore, more commonly used antineoplastic drugs were more likely to be used OL.

## Introduction

Cancer is a leading cause of morbidity and mortality worldwide. Cancer is the second leading cause of death globally, and was responsible for an estimated 9.6 million deaths in 2018, and about 1 in 6 deaths are due to cancer. Approximately 70% of deaths from cancer occur in low- and middle-income countries (WHO, 2018). In the United States, 1.7 million new cancer cases and 0.6 million cancer deaths were projected to occur in 2019 (Siegel et al., 2019). In China, increased incidence of cancer is a major public health problem. Approximately 4.3 million new cancer cases and 2.9 million new cancer deaths occurred in China in 2018 (Chen et al., 2016; Feng et al., 2019). In addition, the survival rate of patients with malignancies has increased. Advances and improvements in diagnostic and therapeutic strategies have resulted in better control, and reduced death rates, related to cancer in countries such as the United States and Europe (De Angelis et al., 2014; Ferlay et al., 2015; WHO, 2018). However, low- and middle-income countries such as China have not experienced this reduction in death rates. Compared to the United States and United Kingdom, China has a lower cancer incidence, but a 30–40% higher cancer mortality rate (Feng et al., 2019). The higher mortality rate in China is a result of a lower diagnosis rate of early stage cancers and non-uniform clinical treatment strategies across regions.

Antineoplastic drugs are widely used to treat cancer (also known as malignancy). However, effective treatments for rare malignancies have not been developed (Saiyed et al., 2017b). Patients with advanced malignancies are more likely to accept the greater risk associated with new therapies. Off-label (OL) drug use (or unlabeled use) is use that is not included in the indications or dosing regimens listed in the approved labeling (Ling-li et al., 2012). Off-label drug use is controversial, but has shown some efficacy for treatment of malignancies. In clinical practice, OL use for treatment of advanced malignancies is common (Herrero Fernandez et al., 2019). Off-label drug use is particularly common for treatment of malignancies due to the existence of numerous cancer subtypes, difficulties involved in performing clinical trials, rapid diffusion of preliminary results, and delays in approval of new drugs by regulatory bodies (Lerose et al., 2011; Herbrand et al., 2019; Mascolo et al., 2020). Therefore, OL use of antineoplastic drugs to treat malignancies is prevalent.

The present study analyzed OL use of antineoplastic drugs for treatment of malignancies in China using data from the Nationwide Medical Insurance database from 2008 to 2010. This was the first study to evaluate OL use at the national level. In addition, this study contributed data from a low-to-middle-income economy (China). Several descriptive studies of OL use for treatment of malignancies have been performed in developed countries. Twenty percent of antineoplastic drugs prescribed to treat malignancies were OL in Italy (Lerose et al., 2011), 27.2% were OL in Switzerland (Joerger et al., 2014), and 35% were OL in Australia (Mellor et al., 2009). Our study characterized OL use of antineoplastic drugs for treatment of malignancies, and described the extent and pattern of OL use. Even though some of the OL indications in 2010 are now accepted practice–that is no longer OL use a few years later, but this study contributed to suggest future strategies that find new use of the old drugs through the OL use. Since the fact that expenditure in oncology medicines is increasingly rapidly, OL use is a more cost-effective choice. It is meaningful to undertake the study to assess the extent of OL use at the time to set a baseline and use the findings to suggest future strategies. We also documented the rationale for OL use. The goal of this study was to provide a basis for development of policies and treatment strategies for OL use of antineoplastic drugs.

## Materials and Methods

### Data Source

Data was acquired from the China Health Insurance Research Association (CHIRA) claims database, which contains a sample of inpatient visits of individuals with Urban Resident Basic Medical Insurance (URBMI) and Urban Employee Basic Medical Insurance (UEBMI) from 2008 to 2010. Urban Resident Basic Medical Insurance is public health insurance for urban non-working residents that covers children, adolescents, college students, elderly individuals, and individuals with disabilities. Urban Employee Basic Medical Insurance covers the working population (public and private sectors) in urban China. Therefore, URBMI and UEBMI are the two largest public insurance providers for urban residents, and cover nearly all urban populations in China. In 2010, the total number of enrollees in URBMI and UEBMI was 432.06 million^1^, which accounted for approximately 65% of the urban population in China^2^. It would be great to expand the data to longer years. However, such data is not available from our provider. A sample from 2008 to 2010 is the latest data available to us, and as far as we know, our data is the only source that covers a wide range of cities regarding inpatient visits and used to evaluate the OL use of antineoplastic drugs for treatment of malignancies. All data generated or analyzed during this study are included in this published article and its Supplementary Material information files. The studies involving human participants are reviewed and approved by Office of Biomedical Ethics Committee of Peking University (IRB00001052–19007).

### Study Sample

Claims submitted to CHIRA were drawn from a few representative cities from the province level Healthcare Security Bureaus. Sampling rates were 10% for county level cities, 5% for prefecture level cities (non-provincial capital cities) and municipalities, and 2% for provincial capital cities. Each record contains information on diagnosis (based on the World Health Organization International Classification of Diseases and Related Health Problems (ICD-10), version 2010^3^), treatment, medication, medical expense, and gender and birth date of the patient.

We used SQL Server 2005 and Network Analysis to select the discharge diagnostic codes (C00-C97) recognized as malignancies to investigate off-label use of antineoplastic drugs to treat malignancies.

### Determination of OL use

Use of drugs for indications not reflected in the package insert published by the National Medical Products Administration at the time of prescription is considered OL use (Seetasith et al., 2017; Fernandez et al., 2019). A summary of results that matched ICD-10 diagnostic codes and the indications included in the package inserts are provided in Supplementary File S1. This standard is used worldwide for general epidemiology, health management, and clinical analysis (Deferio et al., 2018; Nittari et al., 2018).

### Analysis

Descriptive analysis was used to determine the extent of OL use. The ratio of OL to total antineoplastic drug use frequency (DUF) was used to determine the extent of OL use (Prendergast et al., 2013). We also calculated the OL use rate of antineoplastic drugs for treatment of malignancies, and constructed an OL use spectrum for commonly used antineoplastic drugs.

**FIGURE 1:**
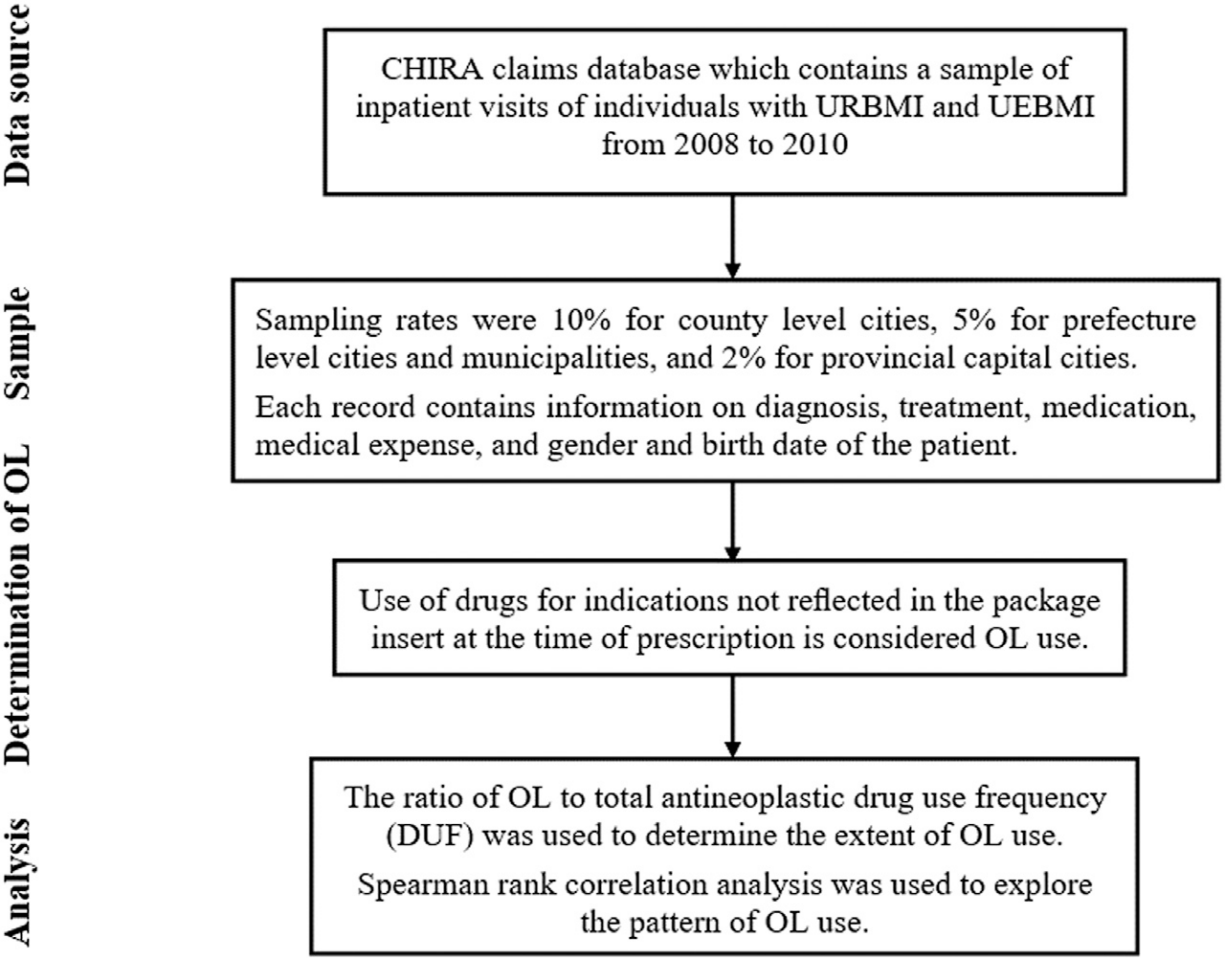
Flowchart of the study design.

Spearman rank correlation analysis was used to explore the pattern of OL use. First, we analyzed the correlation between the disease constituent ratio^4^ (DCR) and the average OL use rate of antineoplastic drugs for treatment of various malignancies. Then, we analyzed the correlation between the average OL use rate of antineoplastic drugs and the number of malignancies these drugs were used to treat. Please see Figure 1 for more details of the study design.

## Results

### Characteristics of the Sample

In this study, 51,382 patients with malignancies who were taking 36 commonly used antineoplastic drugs (frequency of usage above 200) were retrospectively studied. Among 36 commonly used antineoplastic drugs, only 24 antineoplastic drugs could be accurately identified as OL use (the indications of the remaining 12 drugs include a variety of uncertain tumors or the symptoms common to a variety of uncertain tumors). The 24 antineoplastic drugs were found to be off-label used in 77 categories of malignancies (Supplementary File S2).

### Extent of OL Drug Use

**FIGURE 2:**
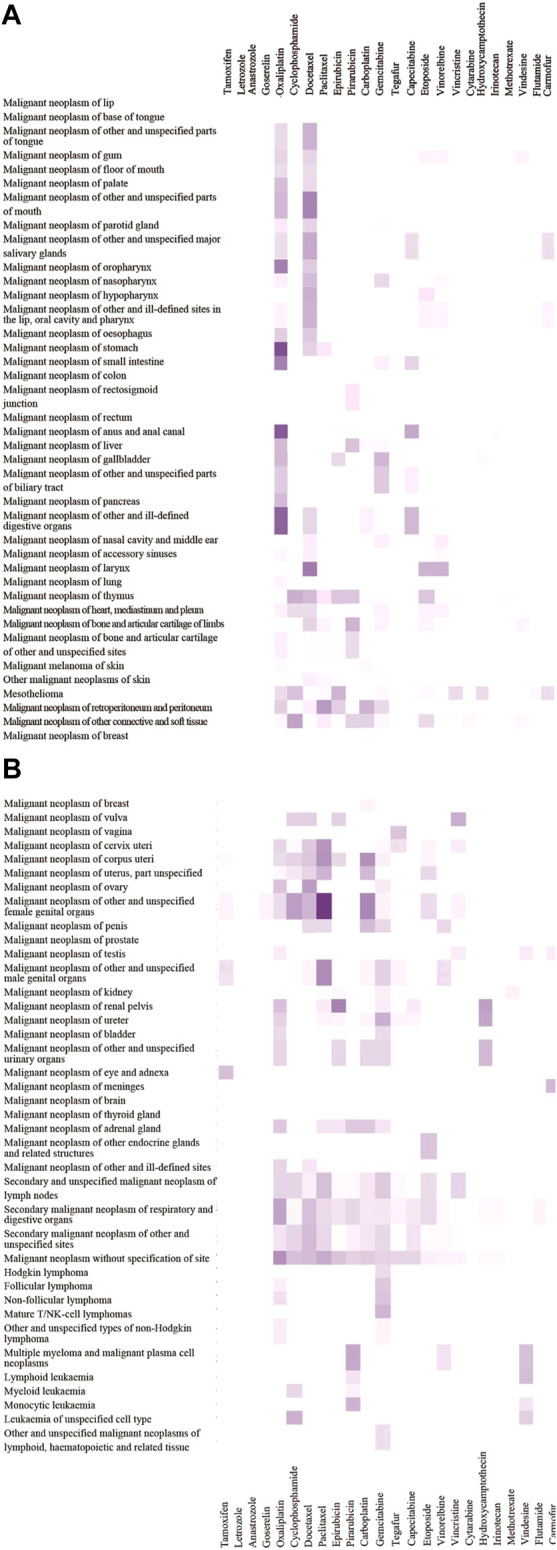
OL use spectrum of 24 antineoplastic drugs in 77 malignancies.The depth of color scale represents the extent of OL use rates (range 0–40%). The malignancies are arranged from top to bottom according to ICD codes (C00-C96). Antineoplastic drugs are sorted in descending order from left to right based on total drug use frequency.

We calculated the ratio of OL drug use frequency (OL-DUF) to total antineoplastic DUF to determine the extent of OL use (Table 1). The results showed that OL use of antineoplastic drugs to treat malignancies was common. Twenty commonly used antineoplastic drugs (ICD encoded as L01) were used OL for 10–61% of cases, and four commonly used endocrine therapy antineoplastic drugs (ICD encoded as L02) were used OL for 10–19% of cases. The highest ratios of OL drug use were those for oxaliplatin (42%), docetaxel (25%), paclitaxel (15%), carboplatin (18%), gemcitabine (20%), vinorelbine (10%), hydroxycamptothecine (26%), irinotecan (27%), vindesine (28%), floxuridine (24%), and carmofur (28%). We constructed an OL use spectrum for 24 commonly used antineoplastic drugs used to treat 77 malignancies. (Figures 2A,B).

**TABLE 1:** Ratio of OL use frequency to total antineoplastic drug use frequency.

Generic name	DUF	OL-DUF	DUF (in unspecified malignancies)	Ratio (%)	Ratio (excludes unspecified malignancies) (%)
Endocrine therapy antineoplastic drug (ICD encoded as L02)					
Tamoxifen	515	97	62	19	7
Letrozole	447	43	32	10	2
Goserelin	200	25	14	13	6
Anastrozole	209	27	23	13	2
Mean	−	−	−	13	4
Antineoplastic drug (ICD encoded as L01)					
Oxaliplatin	5768	3494	1062	61	42
Cyclophosphamide	3599	769	536	21	6
Docetaxel	3421	1441	585	42	25
Paclitaxel	3026	1216	754	40	15
Epirubicin	2826	608	513	22	3
Pirarubicin	2138	799	347	37	21
Carboplatin	2022	771	410	38	18
Gemcitabine	1957	731	334	37	20
Tegafur	1883	381	320	20	3
Capecitabine	1783	476	315	27	9
Etoposide	1353	260	154	19	8
Vinorelbine	1022	230	129	23	10
Vincristine	948	191	135	20	6
Cytarabine	704	70	64	10	1
Hydroxycamptothecine	567	245	98	43	26
Irinotecan	516	241	104	47	27
Methotrexate	452	55	35	12	4
Vindesine	415	179	62	43	28
Fluorouracil	339	144	62	42	24
Carmofur	334	100	5	30	28
Mean	−	−	−	32	16

DUF, drug use frequency; OL-DUF, off-label drug use frequency; DUF (in unspecified malignancies), drug use frequency for unspecified malignancies; Ratio, the ratio of off-label drug use frequency to total antineoplastic drug use frequency; Ratio (exclude unspecified malignancies), the ratio of off-label drug use frequency to antineoplastic drug use frequency, excluding use for unspecified malignancies.

### Pattern of OL Drug Use

**FIGURE 3:**
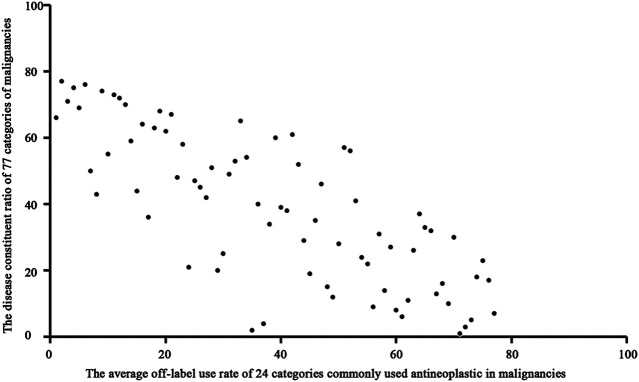
Scatter diagram of the average OL use rate of antineoplastic drugs used to treat malignancies.

**FIGURE 4:**
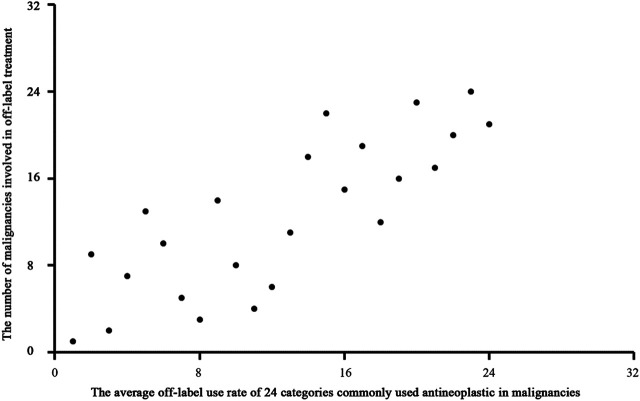
Scatter diagram of the average OL use rate of antineoplastic drugs and the number of malignancies treated using OL drugs.

We used Spearman rank correlation analysis to evaluate patterns of OL use. The results showed a significantly negative correlation (correlation coefficient = −0.778, P = 0.000) between the disease constituent ratio (DCR) and the average OL use rate of antineoplastic drugs for treatment of various malignancies. (Figure 3). The results also showed a significant positive correlation (correlation coefficient = 0.797, *P* = 0.000) between the average OL use rate of antineoplastic drugs and the number of malignancies treated using OL drugs (Figure 4).

## Discussion

The results of this study indicated that OL use of antineoplastic drugs is common in clinical practice across China, which is consistent with the results of other studies (Saiyed et al., 2017a; De Giorgi et al., 2018; Smieliauskas et al., 2018). These results suggested that increased attention should be given to OL use of antineoplastic drugs for treatment of malignancies.

The OL use spectrum for commonly used antineoplastic drugs for treatment of malignancies constructed in this study showed a significant negative correlation between the disease constituent ratio and the average OL use rate of antineoplastic drugs for treatment of various malignancies. This result suggested that uncommon malignancies were more likely to be treated with OL antineoplastic drugs. Uncommon malignancies often do not have standardized treatment courses, and many clinicians may not have experience with treating these cancers. In addition, there was a significant positive correlation between the average OL use rate of antineoplastic drugs and the number of malignancies treated with OL drugs. These results suggested that commonly used antineoplastic drugs were more likely to be used OL to treat malignancies. This may have resulted from clinician familiarity with these drugs. Oxaliplatin is used to treat colorectal cancer, and it is commonly used to treat gastric cancer (42% OL use in this study). Oxaliplatin is the recommended first-line treatment for colorectal and gastric cancer according to the National Comprehensive Cancer Network (NCCN^®^) guidelines, which provides justification for OL use of oxaliplatin to treat gastric cancer. However, oxaliplatin was not recommended by the NCCN^®^ for preoperative gastric cancer treatment until 2013. Our study showed that oxaliplatin was the most commonly used antineoplastic drug to treat gastric cancer in China from 2008 to 2010. Other studies also showed that oxaliplatin was commonly used to treat patients with gastric cancer during this period in China (Liu et al., 2007; Liu et al., 2008; Park et al., 2008; Jiang et al., 2011; Bang et al., 2012) and worldwide (Barone et al., 2007; Park et al., 2008; Koizumi et al., 2010; Lordick et al., 2010; Montagnani et al., 2011). These results indicated that commonly used antineoplastic drugs are often used OL to treat malignancies.

In conclusion, this study showed that OL use of antineoplastic drugs to treat malignancies was prevalent in China from 2008 to 2010. Furthermore, OL use of antineoplastic drugs was more common for treatment of uncommon malignancies. In addition, more commonly used antineoplastic drugs were more likely to be used OL.

It is meaningful to undertake the study to assess the extent of OL use at the time to suggest future traditional drug discovery strategies. Discover new use of the old drugs through the OL use is a more cost-effective future strategy This strategy is highly efficient, time saving, low-cost and minimum risk of failure. It maximizes the therapeutic value of a drug and consequently increases the success rate, which not only provides an effective alternative approach to traditional drug discovery process, but also offers a less expensive treatment plan for patients.

### Our Study Suffered From Several Limitations

To measure OL use, we selected the first three digits of the ICD code for comparison with the labeled indications of each drug. Use was considered on-label for the disease subcategories under each three-digit ICD code. This may have resulted in an underrepresentation of OL use. The current empirical results cannot rule out more interpretations about the conditions of patients’ choice, since the characteristics of the patients submitted for chemotherapy were not available due to the limitation of data.

In addition, the data used in our study were collected from 2008 to 2010, which may not represent current OL use patterns for treatment of malignancies. However, no relevant studies have evaluated OL use of antineoplastic drugs for treatment of malignancies at the national level. Furthermore, the spectra of malignancies and treatment strategies have remained stable in China (Chen et al., 2016). Even though some treatments that were OL in 2010 are now no longer off-label 10°years later. For instance, capecitabine gets approved for gastric adenocarcinoma in 2018. But there are few such examples, which calls for speeding up the drug approval process in China. Propranolol, a non-selective beta-blocker, is an example of a well-known medicine that was reformulated for use in children with proliferating hemangiomas (Léauté-Labrèze et al., 2015). However, to this date (October 2020), it has not been granted in China. Therefore, our study may adequately describe OL use of antineoplastic drugs for treatment of malignancies, and may serve as a scaffold for future studies. Even though some of the OL indications in 2010 are now accepted practice. However, undertook the study to assess the extent of OL use at the time to set a baseline and use the findings to suggest future strategies.

Finally, our study did not evaluate the rationale for OL use of drugs. Further studies should evaluate the rationale for OL use of antineoplastic drugs to treat malignancies by referencing relevant standard treatment guidelines and clinical experience. OL use confronts some clinical and legal risks. OL prescribing is legal in the United States and in many other countries (Verbaanderd et al., 2020). However, there are no standardized regulations on OL use in China, and the law does not specify whether OL use is illegal. Therefore, Medical institutions take the responsibility for regulating and monitoring the OL use (Wu and Wu, 2014). describes a strategy to ensure oversight of patient safety, and prospectively assess efficacy for OL use, and provides a mechanism for the evolution and promotion of standards of medical care for this situation. Furthermore, not all countries support the reimbursement of OL use, so some OL treatments could be unaffordable to patients in China. There are some mature international experience waiting to be practiced to address inappropriate prescribing (Nayroles et al., 2017). This study provided the basis for further research to assess the impact of OL use, and may contribute to development of policies for OL use.

## Data Availability

The original contributions presented in the study are included in the article/Supplementary Material, further inquiries can be directed to the corresponding author.

## Footnotes

1 Please refer to page 74 of http://www.chinacdc.cn/tjsj/gjwstjsj/201205/W020120517331408782774.pdf.

2 The total population living in urban area in China in 2010 was 665.58 million, according to population census in 2010.

3 Please refer to World Health Organization. ICD-10 version 2010 website [Internet].

4 Disease constituent ratio: Since the total number of samples from different regions is not proportional to the total residents, it is not meaningful to compare the number of cases of malignancies among different regions. In order to make the best use of the existing data and understand the geographical distribution characteristics of malignancies, we introduce the concept of disease composition ratio. The proportion of the number of patients with a certain malignancy in the total number of patients in a certain region is defined as the constituent ratio of this malignancies in this region.
