# Supplementary material for: Off-Label Use of Antineoplastic Drugs to Treat Malignancies: Evidence From China Based on a Nationwide Medical Insurance Data Analysis
Source: Front Pharmacol. 2021 Apr 8;12:616453. doi: 10.3389/fphar.2021.616453 (PMC8060556; doi:10.3389/fphar.2021.616453)
Supplement: Supplementary file 1 [file table1.docx]

**Supplementary File 1**

**Table A1**. List of International Classification of Diseases for Malignancies

| ICD code | Malignancy |
| --- | --- |
| C00-C14 | Malignant neoplasms of lip, oral cavity, and pharynx |
| C00 | Malignant neoplasm of lip |
| C01 | Malignant neoplasm of base of tongue |
| C02 | Malignant neoplasm of other and unspecified parts of tongue |
| C03 | Malignant neoplasm of gum |
| C04 | Malignant neoplasm of floor of mouth |
| C05 | Malignant neoplasm of palate |
| C06 | Malignant neoplasm of other and unspecified parts of mouth |
| C07 | Malignant neoplasm of parotid gland |
| C08 | Malignant neoplasm of other and unspecified major salivary glands |
| C09 | Malignant neoplasm of tonsil |
| C10 | Malignant neoplasm of oropharynx |
| C11 | Malignant neoplasm of nasopharynx |
| C12 | Malignant neoplasm of piriform sinus |
| C13 | Malignant neoplasm of hypopharynx |
| C14 | Malignant neoplasm of other and ill-defined sites in the lip, oral cavity, and pharynx |
| C15-C26 | Malignant neoplasms of digestive organs |
| C15 | Malignant neoplasm of esophagus |
| C16 | Malignant neoplasm of stomach |
| C17 | Malignant neoplasm of small intestine |
| C18 | Malignant neoplasm of colon |
| C19 | Malignant neoplasm of rectosigmoid junction |
| C20 | Malignant neoplasm of rectum |
| C21 | Malignant neoplasm of anus and anal canal |
| C22 | Malignant neoplasm of liver and intrahepatic bile ducts |
| C23 | Malignant neoplasm of gallbladder |
| C24 | Malignant neoplasm of other and unspecified parts of biliary tract |
| C25 | Malignant neoplasm of pancreas |
| C26 | Malignant neoplasm of other and ill-defined digestive organs |
| C30-C39 | Malignant neoplasms of respiratory and intrathoracic organs |
| C30 | Malignant neoplasm of nasal cavity and middle ear |
| C31 | Malignant neoplasm of accessory sinuses |
| C32 | Malignant neoplasm of larynx |
| C33 | Malignant neoplasm of trachea |
| C34 | Malignant neoplasm of bronchus and lung |
| C37 | Malignant neoplasm of thymus |
| C38 | Malignant neoplasm of heart, mediastinum, and pleura |
| C39 | Malignant neoplasm of other and ill-defined sites in the respiratory system and intrathoracic organs |
| C40-C41 | Malignant neoplasms of bone and articular cartilage |
| C40 | Malignant neoplasm of bone and articular cartilage of limbs |
| C41 | Malignant neoplasm of bone and articular cartilage of other and unspecified sites |
| C43-C44 | Melanoma and other malignant neoplasms of skin |
| C43 | Malignant melanoma of skin |
| C44 | Other malignant neoplasms of skin |
| C45-C49 | Malignant neoplasms of mesothelial and soft tissue |
| C45 | Mesothelioma |
| C46 | Kaposi sarcoma |
| C47 | Malignant neoplasm of peripheral nerves and autonomic nervous system |
| C48 | Malignant neoplasm of retroperitoneum and peritoneum |
| C49 | Malignant neoplasm of other connective and soft tissue |
| C50-C50 | Malignant neoplasm of breast |
| C50 | Malignant neoplasm of breast |
| C51-C58 | Malignant neoplasms of female genital organs |
| C51 | Malignant neoplasm of vulva |
| C52 | Malignant neoplasm of vagina |
| C53 | Malignant neoplasm of cervix uteri |
| C54 | Malignant neoplasm of corpus uteri |
| C55 | Malignant neoplasm of uterus, part unspecified |
| C56 | Malignant neoplasm of ovary |
| C57 | Malignant neoplasm of other and unspecified female genital organs |
| C58 | Malignant neoplasm of placenta |
| C60-C63 | Malignant neoplasms of male genital organs |
| C60 | Malignant neoplasm of penis |
| C61 | Malignant neoplasm of prostate |
| C62 | Malignant neoplasm of testis |
| C63 | Malignant neoplasm of other and unspecified male genital organs |
| C64-C68 | Malignant neoplasms of urinary tract |
| C64 | Malignant neoplasm of kidney, except renal pelvis |
| C65 | Malignant neoplasm of renal pelvis |
| C66 | Malignant neoplasm of ureter |
| C67 | Malignant neoplasm of bladder |
| C68 | Malignant neoplasm of other and unspecified urinary organs |
| C69-C72 | Malignant neoplasms of eye, brain and other parts of central nervous system |
| C69 | Malignant neoplasm of eye and adnexa |
| C70 | Malignant neoplasm of meninges |
| C71 | Malignant neoplasm of brain |
| C72 | Malignant neoplasm of spinal cord, cranial nerves and other parts of central nervous system |
| C73-C75 | Malignant neoplasms of thyroid and other endocrine glands |
| C73 | Malignant neoplasm of thyroid gland |
| C74 | Malignant neoplasm of adrenal gland |
| C75 | Malignant neoplasm of other endocrine glands and related structures |
| C76-C80 | Malignant neoplasms of ill-defined, secondary, and unspecified sites |
| C76 | Malignant neoplasm of other and ill-defined sites |
| C77 | Secondary and unspecified malignant neoplasm of lymph nodes |
| C78 | Secondary malignant neoplasm of respiratory and digestive organs |
| C79 | Secondary malignant neoplasm of other and unspecified sites |
| C80 | Malignant neoplasm without specification of site |
| C81-C96 | Malignant neoplasms, stated or presumed to be primary, of lymphoid, hematopoietic, and related tissue |
| C81 | Hodgkin lymphoma |
| C82 | Follicular lymphoma |
| C83 | Non-follicular lymphoma |
| C84 | Mature T/NK-cell lymphomas |
| C85 | Other and unspecified types of non-Hodgkin lymphoma |
| C86 | Other specified types of T/NK-cell lymphoma |
| C88 | Malignant immunoproliferative diseases |
| C90 | Multiple myeloma and malignant plasma cell neoplasms |
| C91 | Lymphoid leukemia |
| C92 | Myeloid leukemia |
| C93 | Monocytic leukemia |
| C94 | Other leukemias of specified cell type |
| C95 | Leukemia of unspecified cell type |
| C96 | Other and unspecified malignant neoplasms of lymphoid, hematopoietic and related tissue |
| C97-C97 | Malignant neoplasms of independent (primary)multiple sites |
| C97 | Malignant neoplasms of independent (primary) multiple sites |
