# Supplementary material for: Off-Label Use of Antineoplastic Drugs to Treat Malignancies: Evidence From China Based on a Nationwide Medical Insurance Data Analysis
Source: Front Pharmacol. 2021 Apr 8;12:616453. doi: 10.3389/fphar.2021.616453 (PMC8060556; doi:10.3389/fphar.2021.616453)
Supplement: Supplementary file 2 [file table2.docx]

**Supplementary File 2**

**Table A2**. Approved Indications for 24 Categories Antineoplastic Drugs (Frequency of Usage Above 200)

| Generic name | Frequency of usage | Off-label indications in the package insert and corresponding ICD-10 codes |
| --- | --- | --- |
| L01 Antineoplastics | | |
| Oxaliplatin | 5768 | Malignant neoplasm of colon, Malignant neoplasm of rectosigmoid junction, Malignant neoplasm of rectum (C18-20) |
| Cyclophosphamide | 3599 | Rhabdomyosarcoma C63/76, Osteosarcoma C41/40, Lymphoma(malignant) C81-88/96, Lymphoid leukemia C91/94, Malignant neoplasm of bronchus and lung C34, Malignant neoplasm of breast C50, Malignant neoplasm of testis C62, Malignant neoplasm of ovary C56, Malignant neoplasm of nasopharynx C11, Neuroblastoma C30/72/74, Multiple myeloma C90, Laryngeal neoplasms (head-neck neoplasms) C00-C14/30-32/39/47/69-73/75-76 |
| Docetaxel | 3421 | Malignant neoplasm of breast C50, Malignant neoplasm of bronchus and lung C34, Malignant neoplasm of prostate C61 |
| Paclitaxel | 3026 | Other and unspecified types of non-Hodgkin lymphoma C85, Kaposi sarcoma C46, Malignant neoplasm of bronchus and lung C34, Malignant neoplasm of esophagus C15, Malignant neoplasm of breast C50, Malignant neoplasm of ovary C56, Seminoma C62, Head-neck neoplasms C00-C14/30-32/39/47/69-73/75-76 |
| Epirubicin | 2826 | Leukemia C90-95, Lymphoma(malignant) C81-88/96, Malignant neoplasm of esophagus C15, Malignant neoplasm of stomach C16, Malignant neoplasm of colon, Malignant neoplasm of rectosigmoid junction, Malignant neoplasm of rectum (C18-20), Malignant neoplasm of liver C22, Malignant neoplasm of pancreas C25, Malignant neoplasm of bronchus and lung C34, Malignant neoplasm of breast C50, Malignant neoplasm of ovary C56, Malignant neoplasm of bladder C67, Multiple myeloma C90, Malignant melanoma of skin C43, Sarcoma-soft tissue (C22/38/40/41/46/49/55/63/70/76/79/81/82/83/85/90/92/96) |
| Pirarubicin | 2138 | Malignant neoplasm of stomach C16, Malignant neoplasm of breast C50, Lymphoma(malignant) C81-88/96, Leukemia of unspecified cell type C95, Malignant neoplasms of female genital organs (C51-C58), Malignant neoplasms of male genital organs (C60-C63), Malignant neoplasms of urinary tract (C64-C68), Head-neck neoplasms C00-C14/30-32/39/47/69-73/75-76 |
| Carboplatin | 2022 | Head-neck neoplasms C00-C14/30-32/39/47/69-73/75-76 Lymphoma(malignant) C81-88/96, Malignant neoplasm of ovary C56, Malignant neoplasm of bronchus and lung C34, Malignant neoplasm of testis C62, Malignant neoplasm of cervix uteri C53, Malignant neoplasm of bladder C67, Malignant neoplasm of esophagus C15, Seminoma C62, Mesothelioma C45 |
| Gemcitabine | 1957 | Malignant neoplasm of breast/ Malignant neoplasm of bronchus and lung/ Malignant neoplasm of pancreas (C50/34/25) |
| Tegafur | 1883 | Gastrointestinal neoplasms C15-22/26, Malignant neoplasm of breast C50, Malignant neoplasm of bronchus and lung C34, Malignant neoplasm of bladder C67, Malignant neoplasm of prostate C61, Malignant neoplasm of kidney C64,  Head-neck neoplasms C00-C14/30-32/39/47/69-73/75-76 |
| Capecitabine | 1783 | Malignant neoplasm of stomach/Malignant neoplasm of colon, Malignant neoplasm of rectosigmoid junction, Malignant neoplasm of rectum/Malignant neoplasm of breast (C16/18-20/50) |
| Etoposide | 1353 | Malignant ovarian germ cell tumor †, Lymphoma(malignant) C81-88/96, Leukemia C90-95, Rhabdomyosarcoma C63/76, Malignant neoplasm of stomach C16, Malignant neoplasm of esophagus C15, Neuroblastoma C30/72/74, Malignant neoplasm of bronchus and lung C34, Malignant neoplasm of ovary C56, Malignant neoplasm of testis C62 |
| Vinorelbine | 1022 | Malignant neoplasm of bronchus and lung/ Malignant neoplasm of breast/ Malignant neoplasm of ovary/ Lymphoma(malignant) (C34/50/56/81-88/96) |
| Vincristine | 948 | Malignant neoplasm of bronchus and lung, Malignant neoplasm of breast, Sarcoma-soft tissue, Neuroblastoma, Ovarian germ cell tumor*, Ewing's sarcoma, Nephroblastoma, Malignant melanoma of skin, Multiple myeloma  (C30/34/38/40/41/43/46/49/50/55/63/64/70/72/74/76/79) Leukemia of unspecified cell type/ Hodgkin lymphoma/ Lymphoma(malignant) (C81-96), Gastrointestinal neoplasms (C15-21/26) |
| Cytarabine | 704 | Leukemia C90-95, lymphoma(C81-96) |
| Hydroxycamptothecin | 567 | Leukemia C90-95, Malignant neoplasm of liver/ Malignant neoplasm of stomach/ Malignant neoplasm of bladder/ Malignant neoplasm of rectum/ Head-neck neoplasms (C00-14/16/20/22/30-32/39/47/67/69-73/75-76) |
| Irinotecan | 516 | Malignant neoplasm of colon, Malignant neoplasm of rectosigmoid junction, Malignant neoplasm of rectum (C18-20) |
| Methotrexate | 452 | Malignant neoplasm of breast, Malignant neoplasm of ovary, Malignant neoplasm of cervix uteri, Chorioadenoma, Malignant neoplasm of testis, Malignant neoplasm of bronchus and lung, Head-neck neoplasms, Psoriasis**,** Leukemia, Lymphoma, Lymphoma(malignant), Granuloma fungoides, Sarcoma-soft tissue (C00-14/22/30-32/34/38/39/40/41/46/47/49/50/53/55/56/62/63/69-73/75/76/79/81-96/) [Multiple myeloma](http://www.youdao.com/w/multiple%20myeloma/#keyfrom=E2Ctranslation) C92, Central nervous system neoplasms (C70-72) |
| Vindesine | 415 | Malignant neoplasm of bronchus and lung, Lymphoma(malignant), Malignant neoplasm of breast, Malignant neoplasm of esophagus, Malignant melanoma of skin (C15, C34, C50, C81-88/96, C43) |
| Fluorouracil | 339 | Malignant neoplasm of liver, Malignant neoplasm of rectum, Malignant neoplasm of esophagus, Malignant neoplasm of stomach, Malignant neoplasm of breast, Malignant neoplasm of bronchus and lung (C22/20/15/16/34/50) |
| Carmofur | 334 | Gastrointestinal neoplasms (Malignant neoplasm of esophagus, Malignant neoplasm of stomach, Malignant neoplasm of colon, Malignant neoplasm of rectum), Malignant neoplasm of breast (C15-21/26/50) |
| L02 Endocrine therapy drugs | | |
| Tamoxifen | 515 | Malignant neoplasm of breast C50 |
| Letrozole | 447 | Malignant neoplasm of breast C50 |
| Anastrozole | 209 | Malignant neoplasm of breast C50 |
| Goserelin | 200 | Malignant neoplasm of prostate, Malignant neoplasm of breast, Endometriosis (C61/50) |

† A total of 51,382 patients, which included 1 patient diagnosed with germ cell tumor (C80). The patient was treated with etoposide rather than vincristine.

‡ The underline indicates that there was no appropriate indication in the diagnosis code for patients with cancer (ICD C00-C97).
